# Lossy Infrared Image Compression Based on Wavelet Coefficient Probability Modeling and Run-Length-Enhanced Huffman Coding

**DOI:** 10.3390/s25082491

**Published:** 2025-04-15

**Authors:** Yaohua Zhu, Ya Liu, Yanghang Zhu, Mingsheng Huang, Jingyu Jiang, Yong Zhang

**Affiliations:** 1 Shanghai Institute of Technical Physics, Chinese Academy of Sciences, Shanghai 200083, China; zhuyaohua@mail.sitp.ac.cn (Y.Z.); ly_hnyc@163.com (Y.L.); sc22219026@mail.ustc.edu.cn (Y.Z.); huangmingsheng@mail.ustc.edu.cn (M.H.); jiangjingyu23@mails.ucas.ac.cn (J.J.); 2 University of Chinese Academy of Sciences, Beijing 100049, China; 3 Laboratory of Infrared Detection and Imaging Technology, Chinese Academy of Sciences, Shanghai 200083, China

**Keywords:** image lossy compression, Huffman coding, JPEG2000, infrared line-scanning images, DWT

## Abstract

Infrared line-scanning images have high redundancy and large file sizes. In JPEG2000 compression, the MQ arithmetic encoder’s complexity slows down processing. Huffman coding can achieve O(1) complexity based on a code table, but its integer-bit encoding mechanism and ignorance of the continuity of symbol distribution result in suboptimal compression performance. In particular, when encoding sparse quantized wavelet coefficients that contain a large number of consecutive zeros, the inaccuracy of the one-bit shortest code accumulates, reducing compression efficiency. To address this, this paper proposes Huf-RLC, a Huffman-based method enhanced with Run-Length Coding. By leveraging zero-run continuity, Huf-RLC optimizes the shortest code encoding, reducing the average code length to below one bit in sparse distributions. Additionally, this paper proposes a wavelet coefficient probability model to avoid the complexity of calculating statistics for constructing Huffman code tables for different wavelet subbands. Furthermore, Differential Pulse Code Modulation (DPCM) is introduced to address the remaining spatial redundancy in the low-frequency wavelet subband. The experimental results indicate that the proposed method outperforms JPEG in terms of PSNR and SSIM, while maintaining minimal performance loss compared to JPEG2000. Particularly at low bitrates, the proposed method shows only a small gap with JPEG2000, while JPEG suffers from significant blocking artifacts. Additionally, the proposed method achieves compression speeds 3.155 times faster than JPEG2000 and 2.049 times faster than JPEG.

## 1. Introduction

In practical image lossy compression systems, the framework based on transform–quantization–entropy coding is the most widely used [1,2,3,4,5,6]. However, methods that currently outperform JPEG2000 in compression efficiency typically come at the cost of increased computational complexity. The core of transform coding lies in the elimination of redundant correlations in image data, thus enhancing compression efficiency. Transformation methods in image compression can be divided into linear and non-linear transformations, with the latter commonly employed in deep learning-based image compression methods [7,8,9,10,11]. The activation functions in the network architecture introduce non-linearity, thereby implementing non-linear transformations that extract complex features to achieve higher compression performance. However, deep learning-based image compression methods have higher computational complexity, which limits their application in scenarios where speed is a critical factor. Common linear transform methods include the Karhunen–Loève transform (KLT) [12], Discrete Cosine Transform (DCT) [13,14], and Discrete Wavelet Transform (DWT) [15,16,17,18], with DCT and DWT serving as core transform techniques in the JPEG and JPEG2000 standards, respectively.

In terms of eliminating data correlations, the KLT, as an optimal linear orthogonal transform, can adaptively determine the optimal orthogonal basis based on the statistical properties of the data, minimizing the correlation between data in the new basis coordinate system. It achieves optimal compression in terms of the Mean Squared Error (MSE) [12]. However, the high computational complexity of the KLT limits its use in practical applications.

In contrast, the basis functions of DCT, which are cosine functions, are fixed and independent of the statistical properties of the data, significantly reducing computational complexity. Moreover, DCT can achieve compression performance comparable to that of KLT in most cases, making it a widely used alternative in practical systems. However, as global basis functions, cosine functions span the entire dataset, limiting DCT’s ability to capture local features, such as image edges and textures. Therefore, when processing non-stationary signals, such as images, its redundancy reduction effectiveness is constrained.

To reduce the high computational and storage complexity of performing global DCT on large images, JPEG uses an 8 × 8 block-based DCT. However, under low bitrate conditions, information loss caused by truncated high-frequency components often leads to the blurring of the image and block artifacts.

The DWT employs wavelet functions as basis functions, with commonly used wavelet functions including Haar [19], Daubechies [20], Biorthogonal [21], and Coiflet [22]. Wavelet functions exhibit locality, being nonzero only in a limited region, which allows the DWT to be implemented through a weighted summation (convolution) method within local regions. As a result, the convolution-based DWT can operate directly on the entire image without the need for block-based processing, effectively avoiding block artifacts under low bitrate conditions. The DWT can simultaneously capture both global and local features of an image, making it more suitable for processing non-stationary signals.

Furthermore, the DWT retains both time-domain and frequency-domain information and supports integer lossless transformations, making it more advantageous than the DCT in preserving image details. Its hierarchical decomposition mechanism allows images to be transmitted progressively at different resolution and quality levels, aligning more closely with human visual characteristics. Due to these properties, wavelet transforms are widely used in modern image compression techniques such as EZW (Embedded Zerotree Wavelet), SPIHT (Set Partitioning in Hierarchical Trees), and EBCOT (Embedded Block Coding with Optimal Truncation) [23,24,25].

The Mallat algorithm is a fast DWT that achieves efficient multi-resolution decomposition [26]. This algorithm implements DWT through convolution operations using FIR (Finite Impulse Response) filters. Introduced later, the lifting scheme decomposes the traditional filtering operations in the Mallat algorithm into a series of simple, complementary prediction and update steps, resulting in an efficient and reversible DWT, while also mitigating the boundary effects of convolution [27,28]. The lifting-based DWT is essentially an optimized version of the Mallat algorithm, and mathematically, they are equivalent. The lifting-based DWT is applied in JPEG2000 due to its computational simplicity and efficiency.

An *n*-level wavelet transform decomposition results in 3n+1 subbands, with the low-frequency subband (LL) containing most of the energy and serving as the approximation of the original image, while the high-frequency subbands (LH, HL, HH) contain less energy and represent the details of the image. Although the DWT offers advantages over the DCT, the decomposed subbands, especially the LL subband, may still contain spatial redundancy due to the retention of time-domain information.

Quantization schemes typically include vector quantization (VQ) and scalar quantization (SQ). In high bitrate scenarios, uniform scalar quantization (USQ) is widely used in practical coding systems due to its good rate-distortion performance and simplicity [29]. JPEG uses an 8 × 8 DCT transformation, combined with USQ [30,31,32,33]. However, under low bitrate conditions, the quality of JPEG-compressed images degrades significantly due to the side effects introduced by quantization and block division. In contrast, JPEG2000 uses a lifting-based DWT to transform the entire image, avoiding block artifacts. JPEG2000 uses two wavelet transforms: the Le Gall 5/3 integer wavelet transform for lossless compression, and the Daubechies 9/7 fractional wavelet transform for lossy compression [18]. In lossy compression, JPEG2000 uses dead-zone uniform scalar quantization (DUSQ) [29,34,35] during the quantization stage. This method introduces larger quantization intervals near zero to discard insignificant information, thereby improving compression performance. Infrared line-scanning images, due to the high amount of redundant information they contain, exhibit sparsity in the coefficients of high-frequency subbands after DUSQ, with many coefficients being zero, and the few nonzero coefficients concentrated near zero.

Entropy coding is a key step in image compression, with common methods including arithmetic coding (AC) [36,37,38,39] and Huffman coding [40,41,42,43,44]. JPEG2000 adopts MQ arithmetic coding [45,46], which uses a two-level lookup table with context (CX) and binary decisions (D) for adaptive probability estimation, achieving high compression efficiency. However, this method has high computational complexity. Despite optimizations such as parallel algorithms and hardware acceleration [47,48], its theoretical complexity is higher than that of Huffman coding. In contrast, Huffman coding is more efficient in terms of computational speed. In our previous work, we designed a coding method that could achieve O(1) complexity after the codebook was constructed [49], making it highly suitable for high-speed compression applications.

However, Huffman coding only assigns integer-bit codes and does not consider the continuity of symbol distributions, resulting in low compression efficiency for sparse wavelet coefficients after quantization. Specifically, for high-frequency subbands with large numbers of consecutive zeros, the inaccuracy of Huffman coding with a minimum code length of one bit accumulates, reducing compression efficiency. Furthermore, due to the differences in statistical distributions across various wavelet subbands, traditional Huffman coding often requires frequent recalculation of statistics for constructing the codebook, thereby increasing complexity.

To address the remaining spatial redundancy in wavelet subbands and the inefficiencies of Huffman coding for quantized sparse wavelet coefficients, this paper proposes the following optimization strategies:

1. Wavelet subband redundancy reduction via DPCM:
  to address the spatial redundancy in wavelet subbands caused by the presence of temporal information, Differential Pulse Code Modulation (DPCM) is employed to reduce spatial redundancy.
2. Wavelet coefficient probability modeling for Huffman coding:
  A wavelet coefficient probability model-based Huffman coding scheme is proposed to eliminate the need for frequent symbol statistics and codebook reconstruction across wavelet subbands, thereby reducing computational complexity. Since quantized wavelet coefficients have varying statistical distributions across subbands and quantization levels, traditional Huffman coding requires frequent coefficient statistics and codebook construction, leading to high computational costs. The proposed method establishes a probabilistic model for quantized wavelet coefficients to effectively reduce complexity and improve compression speed.
3. Run-length-enhanced Huffman coding:
  Huffman coding of quantized sparse wavelet coefficients suffers from inefficient code length allocation and disregards symbol distribution continuity, leading to suboptimal compression efficiency. By integrating Run-Length Coding (RLC) into Huffman coding, symbol continuity is exploited to optimize the assignment of the shortest Huffman code, which is one bit, achieving an average code length of less than one bit in sparse scenarios, thus improving the efficiency of Huffman coding.

Experimental results demonstrate that the proposed method outperforms JPEG in terms of compression ratio and decoding speed, achieves higher compression speed compared to JPEG2000, and maintains image quality comparable to JPEG2000 at the same bitrate. Especially under low bitrate conditions, the proposed method maintains a small gap with JPEG2000, while JPEG shows significant blocking artifacts. Speed test results show that the proposed method achieves compression speeds 3.155 times faster than JPEG2000 and 2.049 times faster than JPEG, providing an ideal solution for lossy compression applications that require both compression speed and image quality.

## 2. Proposed Method

### 2.1. DWT and Coefficient Quantization

Infrared images typically exhibit high spatial redundancy. Image transforms can eliminate this redundancy, facilitating subsequent quantization and entropy coding. The Discrete Wavelet Transform (DWT) offers advantages aligned with human visual perception, making it ideal for image compression schemes based on human vision characteristics.

#### 2.1.1. Overview of Lifting-Based DWT

The DWT utilizes wavelet functions as basis functions, which are nonzero only within a localized region. This locality allows the DWT to be efficiently implemented using short convolution kernels, as convolution inherently involves a weighted summation of localized regions of the signal. Furthermore, the DWT implemented via convolution can operate on the entire image without block division, eliminating block effects under low bitrate conditions.

The Mallat algorithm is a fast DWT computation method based on Multi-Resolution Analysis (MRA), which decomposes and reconstructs the signal layer by layer using filter banks. After a single level of DWT decomposition, the image is divided into four subbands: the low-frequency subband (LL) and three high-frequency subbands (LH, HL, HH). In multi-level decomposition, each transformation is applied only to the LL subband of the previous level, thereby enabling the extraction of image features at multiple scales.

The Mallat algorithm performs decomposition using a low-pass filter h[n] and a high-pass filter g[n]. The one-dimensional (1D) transformation formula is as follows.

The decomposition formula for the low-frequency (approximation) component is defined as:

(1) $$a_{j+1}\left[k\right]=\sum_{n}h\left[n-2k\right]a_{j}\left[n\right]$$

The decomposition formula for the high-frequency (detail) component is defined as follows:

(2) $$d_{j+1}\left[k\right]=\sum_{n}g\left[n-2k\right]a_{j}\left[n\right]$$

where: $a_{j}\left[n\right]$ is the low-frequency coefficient at the *j*th level, with *j* = 0 corresponding to the original data. $d_{j+1}\left[k\right]$ is the high-frequency coefficient at the j+1th level. Due to the 2-fold downsampling, the number of coefficients at each level is halved compared to the previous level. Specifically, when using filters aligned at index 0 (e.g., the CDF 9/7 wavelet), the low-pass filter typically retains even-indexed samples while the high-pass filter retains odd-indexed ones, or vice versa, depending on the filter structure and implementation.

The signal reconstruction process involves upsampling, where a zero is inserted between each pair of consecutive samples, followed by low-pass and high-pass reconstruction filtering and summation. This process is defined as follows:

(3) $$a_{j}\left[n\right]=\sum_{k}\tilde{h}\left[n-2k\right]a_{j+1}\left[k\right]+\sum_{k}\tilde{g}\left[n-2k\right]d_{j+1}\left[k\right]$$

where $\tilde{h}\left[n\right]$ and $\tilde{g}\left[n\right]$ represent the filters used for reconstruction.

For a two-dimensional (2D) infrared image X(m,n), the 2D Mallat algorithm is required, which applies high-pass and low-pass filters along both rows and columns. The transformation is defined as follows:

The first step is to perform a 1D wavelet decomposition on each row.

(4) $$A\left(m,k\right)=\sum_{n}h\left[n-2k\right]X\left(m,n\right)$$

(5) $$D\left(m,k\right)=\sum_{n}g\left[n-2k\right]X\left(m,n\right)$$

After the row-wise transformation, the image is decomposed into a low-frequency component A(m,k) and a high-frequency component D(m,k).

In the second step, a 1D wavelet decomposition is applied along the column direction to both A(m,k) and D(m,k), resulting in four subbands:

The LL (Low–Low) subband, which represents the low-frequency approximation component, is defined as follows:

(6) $$LL\left(l,k\right)=\sum_{m}h\left[m-2l\right]A\left(m,k\right)$$

The LH (Low–High) subband, which represents the high-frequency detail in the horizontal direction, is defined as follows:

(7) $$LH\left(l,k\right)=\sum_{m}g\left[m-2l\right]A\left(m,k\right)$$

The HL (High–Low) subband, representing high-frequency details in the vertical direction, is defined as follows:

(8) $$HL\left(l,k\right)=\sum_{m}h\left[m-2l\right]D\left(m,k\right)$$

The HH (High–High) subband, which represents high-frequency detail in the diagonal direction, is defined as follows:

(9) $$HH\left(l,k\right)=\sum_{m}g\left[m-2l\right]D\left(m,k\right)$$

**Figure 1:**
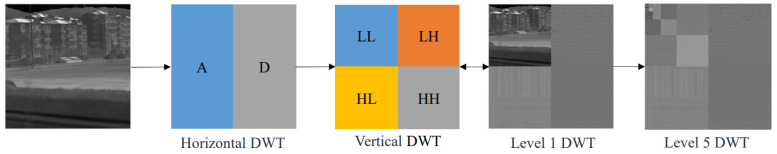
The Mallat decomposition process of an image. The letter A indicates the low-frequency approximation subband, and D indicates the high-frequency detail subbands. Different colors are used to distinguish the subbands for clarity.

The next level of decomposition only requires performing 2D Mallat decomposition on the LL subband. The Mallat decomposition process of an image is shown in Figure 1.

During image reconstruction, the inverse transform is first applied to each column.

(10) $$A\left(m,k\right)=\sum_{l}\tilde{h}\left[m-2l\right]LL\left(l,k\right)+\sum_{l}\tilde{g}\left[m-2l\right]LH\left(l,k\right)$$

(11) $$D\left(m,k\right)=\sum_{l}\tilde{h}\left[m-2l\right]HL\left(l,k\right)+\sum_{l}\tilde{g}\left[m-2l\right]HH\left(l,k\right)$$

Then, the inverse transform is applied to each row to reconstruct the original image.

(12) $$\tilde{X}\left(m,n\right)=\sum_{k}\tilde{h}\left[n-2k\right]A\left(m,k\right)+\sum_{k}\tilde{g}\left[n-2k\right]D\left(m,k\right)$$

The construction of the DWT is flexible, allowing the selection of different wavelet basis functions according to specific applications, with corresponding filter coefficients. In JPEG2000, both Daubechies 9/7 DWT and Le Gall 5/3 DWT are employed, with the Daubechies 9/7 DWT being used for lossy compression. The Daubechies 9/7 DWT includes a low-pass filter with 9 coefficients and a high-pass filter with 7 coefficients. These filter coefficients are carefully computed to achieve efficient image compression performance. The filter coefficients are shown in Table 1 [18].

In the decomposition process, the filters are referred to as Analysis Filter Coefficients, while in the reconstruction process, they are referred to as Synthesis Filter Coefficients, forming a pair of dual filters.

The Mallat algorithm uses FIR filters for convolution calculations, which are sensitive to boundaries and require special handling. In contrast, the lifting-based DWT decomposes the traditional filtering operations in the Mallat algorithm into a series of simple, complementary prediction and update steps, resulting in an efficient and reversible DWT. The lifting-based DWT is also boundary-insensitive and more memory-efficient.

The lifting-based DWT requires the signal x(n) to be split, typically into an odd sequence $x_{o}\left(n\right)$ and an even sequence $x_{e}\left(n\right)$. Prediction and update are the fundamental steps in the lifting process. The differences between various DWTs lie in the number of lifting steps and the parameters used.

In the prediction step, the $x_{e}\left(n\right)$ is used to predict the $x_{o}\left(n\right)$, thus calculating the high-frequency part of the signal, which is defined as follows:

(13) $$d_{n}=x_{o}\left[n\right]-P\left(x_{e}\left[n\right]\right)$$

In the update step, the predicted high-frequency is used to update the $x_{e}\left(n\right)$, thereby calculating the low-frequency part of the signal, which is defined as follows:

(14) $$a_{n}=x_{e}\left[n\right]-U\left(d_{n}\right)$$

where *P* and *U* are the prediction and update operators.

This paper uses the lifting-based Daubechies 9/7 DWT, employing two lifting steps and one scale normalization step. The two lifting steps are defined as follows:

(15) $$d_{n}=x_{o}\left[n\right]+\alpha \left(x_{e}\left[n\right]+x_{e}\left[n+1\right]\right)$$

(16) $$a_{n}=x_{e}\left[n\right]+\beta \left(d_{n}+d_{n+1}\right)$$

(17) $$d_{n}=d_{n}+\gamma \left(a_{n}+a_{n+1}\right)$$

(18) $$a_{n}=a_{n}+\delta \left(d_{n}+d_{n+1}\right)$$

The one scale normalization step is defined as follows:

(19) $$a_{n}=a_{n}\cdot \xi$$

(20) $$d_{n}=d_{n}/\xi$$

**Figure 2:**
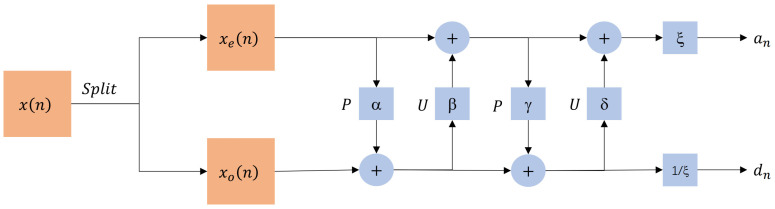
Structure of lifting-based Daubechies 9/7 DWT.

The structure of the lifting-based Daubechies 9/7 DWT is shown in Figure 2.

Scale normalization amplifies the low frequencies by ξ and reduces the high frequencies by ξ, ensuring a reasonable distribution of energy between the low and high frequencies. In JPEG2000, the parameters used are shown in Table 2 [27].

The formulas for decomposition and reconstruction in the lifting scheme are perfectly symmetric, with the parameters remaining unchanged.

#### 2.1.2. Wavelet Coefficients’ Dead-Zone Uniform Scalar Quantization

Different wavelet subbands have different importance and coefficient variance, so each subband’s coefficients need to be quantized with different bit allocation to achieve optimal coding performance [18]. The LL subband contains the approximate information of the image, capturing the majority of the original image’s energy. It defines the overall structure of the image and is highly sensitive to human visual perception. Therefore, a precise quantization is applied to the LL subband to preserve as much visual information as possible. The high-frequency subbands (LH, HL, HH) contain the detailed information of the image, which is less critical. Therefore, coarser quantization can be applied to reduce storage requirements and improve the compression ratio.

In JPEG2000, each subband uses an independent quantization step size, but the quantization step size within the same subband is uniform. The quantization is implemented using a quantization table [18,50]. JPEG2000 adopts Dead-Zone Uniform Scalar Quantization (DUSQ), an improvement of Uniform Scalar Quantization (USQ). This method sets a larger quantization step size near the zero region, removing more non-important information and further increasing the proportion of zero values. Properly setting the dead zone range can effectively reduce the number of output bits from the entropy encoder and improve compression efficiency [34].

**Figure 3:**
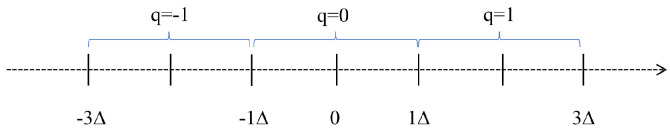
Illustration of USQ. Blue brackets indicate intervals quantized to the same value.

The commonly used USQ formula can be expressed as follows:

(21) $$q\left(c\right)=\left\{\begin{array}{c} 0,c\in [-\Delta ,+\Delta ]\\ n,c\in \left((2n-1)\Delta ,(2n+1)\Delta \right] \end{array}\right.$$

where *c* denotes the data to be quantized, and q(c) represents the quantized value. The quantization step size is 2Δ, and *n* is an integer. The illustration of USQ is shown in Figure 3.

**Figure 4:**
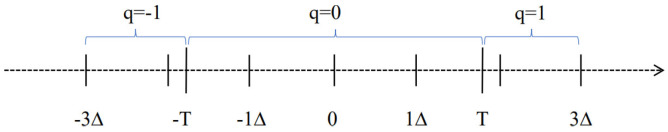
Illustration of DUSQ. Blue brackets indicate intervals quantized to the same value.

The commonly used DUSQ formula can be expressed as follows:

(22) $$q\left(c\right)=\left\{\begin{array}{c} 0,c\in [-T,+T]\\ -1,c\in [-3\Delta ,-T]\\ +1,c\in [T,+3\Delta ]\\ n,c\in \left((2n-1)\Delta ,(2n+1)\Delta \right] \end{array}\right.$$

where *n* is a integer, but n≠±2. The dead zone is [−T,+T]. The illustration of DUSQ is shown in Figure 4.

The wavelet coefficient quantization equation used in this paper was expressed as:

(23) $$q\left(c_{j}\left(k\right)\right)=\left\{\begin{array}{c} 0,\mathrm{if}\;|c_{j}\left(k\right)|<T\\ \mathrm{sign}\left(c_{j}\left(k\right)\right)\cdot \left\lfloor \frac{|c_{j}\left(k\right)|}{\Delta_{j}}\right\rceil ,\;\mathrm{otherwise} \end{array}\right.$$

The dequantization formula was expressed as:

(24) $$\tilde{c_{j}}\left(k\right)=\mathrm{sign}\left(q\left(c_{j}\left(k\right)\right)\right)\cdot \Delta_{j}\cdot q\left(c_{j}\left(k\right)\right)$$

where sign(·) represents the sign function; ⌊·⌉ denotes rounding; $c_{j}\left(k\right)$ is the wavelet coefficient; $\tilde{c_{j}}\left(k\right)$ is the dequantized wavelet coefficient; $q(c_{j}\left(k\right))$ is the quantized coefficient; and $\Delta_{j}=2\Delta$ is the quantization step size, related to the dynamic range of subband *j*, and refers to the quantization table used in JPEG2000 [18,50]. In this paper, T=1.7Δ.

#### 2.1.3. Classification of Quantization Levels

To achieve variable bitrate compression, wavelet coefficients are quantized at different levels, with the quantization level denoted by the parameter *Q*. The quantization formula is given as follows:

(25) $$q\left(c_{j}\left(k\right)\right)=\left\{\begin{array}{c} 0,\mathrm{if}\;|c_{j}\left(k\right)|<T\\ \mathrm{sign}\left(c_{j}\left(k\right)\right)\cdot \left\lfloor \frac{|c_{j}\left(k\right)\cdot Q|}{\Delta_{j}}\right\rceil ,\;\mathrm{otherwise} \end{array}\right.$$

Different quantization levels result in different probability distributions. However, when the quantization levels are close, the differences in probability distributions are relatively small. Therefore, similar quantization levels are grouped into the same quantization category, leading to a graded classification of quantization levels. In the experiment, the parameter *Q* ranged from 0 to 32 and was divided into 5 quantization levels based on the bit length of the parameter *Q*, as shown in Table 3.

**Figure 5:**
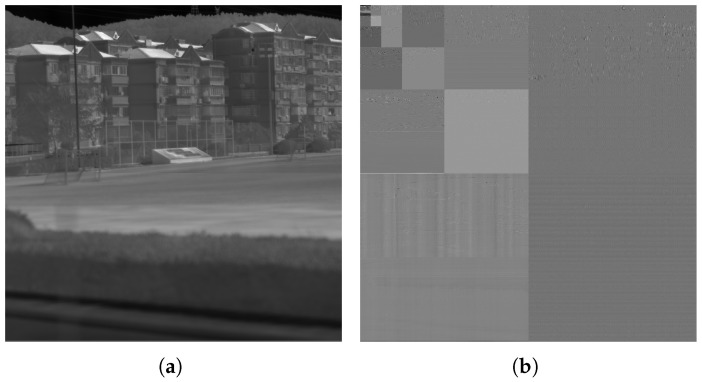
Image A and its five-level DWT. (**a**) Image A. (**b**) Five-level DWT of Image A.

**Figure 6:**
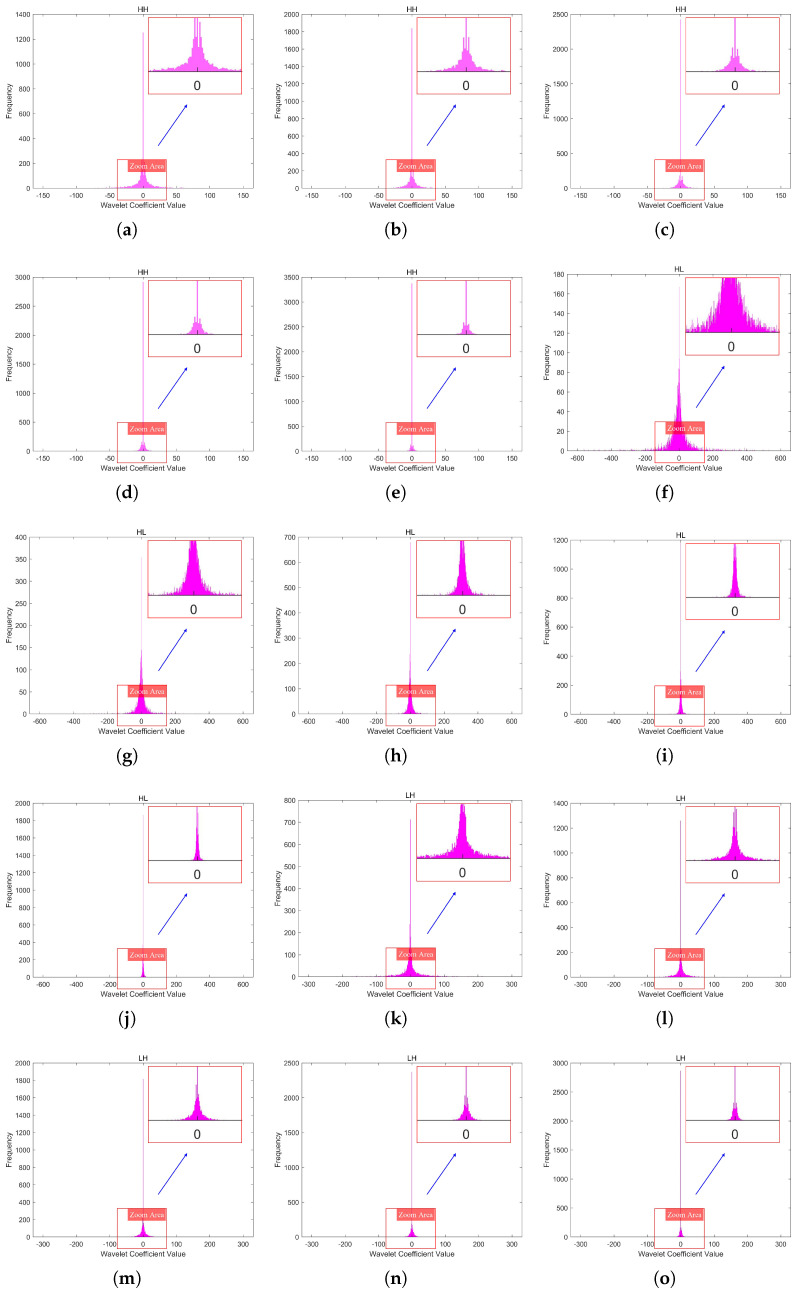
The coefficient histograms of the three high-frequency subbands(LH, HL, HH) in the fifth-level DWT of Image A under different quantization levels. (**a**–**e**) depict the coefficient histograms of the HH subband at the fifth level of Image A, corresponding to quantization levels 1 to 5. (**f**–**j**) depict the coefficient histograms of the HL subband at the fifth level of Image A, corresponding to quantization levels 1 to 5. (**k**–**o**) depict the coefficient histograms of the LH subband at the fifth level of Image A, corresponding to quantization levels 1 to 5.

In this paper, we used a 2048 × 2048 infrared line-scanning image (Image A) as an example, with its five-level DWT shown in Figure 5. The coefficient histograms of the three high-frequency subbands (LH, HL, HH) in the fifth-level DWT of Image A under different quantization levels are shown in Figure 6. It can be observed that as the quantization level increases, the sparsity of the wavelet coefficients intensifies, leading to more zeros.

### 2.2. The Reduction in Spatial Redundancy and Probability Modeling of Quantized Wavelet Subbands

#### 2.2.1. Analysis and Removal of Spatial Redundancy in Quantized Wavelet Subbands

Compared to the DCT, the DWT exhibits superior time–frequency localization properties. While the wavelet decomposition retains additional temporal information, enhancing the retention of image details and edge structures, it also introduces spatial redundancy. As a result, its redundancy reduction efficiency may lead to suboptimal performance compared to the DCT, which more closely approximates the KLT.

The LL subband serves as an approximation of the original image and retains the most spatial redundancy. In contrast, the high-frequency subbands (LH, HL, HH) represent high-frequency details in the horizontal, vertical, and diagonal directions, and exhibit lower spatial redundancy due to their lower information content. Since each subband preserves directional features, directional differential methods such as DPCM can be employed to reduce spatial redundancy.

DPCM was chosen for its simplicity and effectiveness in reducing redundancy in line-scanned images, which helps maintain low algorithm complexity. More sophisticated predictors could improve compression efficiency but would add complexity.

1. LH subband (horizontal details):
  The coefficients exhibit significant variations along the horizontal direction, making row-wise differencing effective for redundancy reduction.
2. HL subband (vertical details):
  The coefficients vary significantly along the vertical direction, making column-wise differencing more suitable for redundancy reduction.
3. LL (low-frequency) and HH (diagonal detail) subbands:
  These subbands lack strong directionality, and both row-wise and column-wise differencing methods can be applied. The optimal approach depends on the specific characteristics of the image.

In this paper, we calculated the original entropy of the subbands at each decomposition level of Image A, and the entropy after the redundancy was reduced by DPCM. The results presented in Table 4 lead to the following conclusions based on the experimental data:

- Infrared line-scanning images typically contain noise [51]; line-scanning images are acquired as the detector scans along the horizontal direction, exhibiting strong inter-column correlation, which results in lower energy and lower entropy in horizontal details (LH) compared to vertical details (HL).
- The HH subband is sensitive to noise because it contains minimal entropy. As a result, the application of differencing operations tends to amplify the noise, making the data distribution more dispersed and increasing the information entropy, which introduces additional redundancy. For example, in the 5th-level HH subband, the original entropy was 5.0752. After applying DPCM in the column and row directions, the entropy increased to 5.8301 and 5.8806, respectively.
- The HL subband still exhibits some inter-column correlation, and column-wise differencing can reduce redundancy, but its effect is not as significant. For instance, in the 5th-level HL subband, applying DPCM along the column direction reduced the information entropy from 7.5204 to 6.9991, achieving a reduction of 0.5231.
- The LH subband removes inter-column correlation, has low energy, and is easily affected by noise. For example, in the 5th-level LH subband, the original entropy was 6.1932. After applying DPCM in the column and row directions, the entropy increased to 6.9936 and 6.5551, respectively.
- The LL subband contains the majority of the image information and exhibits significant spatial redundancy. For example, in the 5th-level LL subband, the original entropy was 9.7194. After applying DPCM in the column and row directions, the entropy decreased to 7.3093 and 8.5251, respectively. The column-wise DPCM achieved a more significant entropy reduction of 2.4101. Additionally, the coefficient distribution of the LL subband shows no regularity; however, after redundancy reduction using DPCM, it approximates a Laplace distribution, which is advantageous for subsequent probabilistic modeling.

Based on the above analysis, this paper employed column-wise DPCM for redundancy reduction only on the LL subband, in order to maintain low algorithmic complexity.

**Figure 7:**
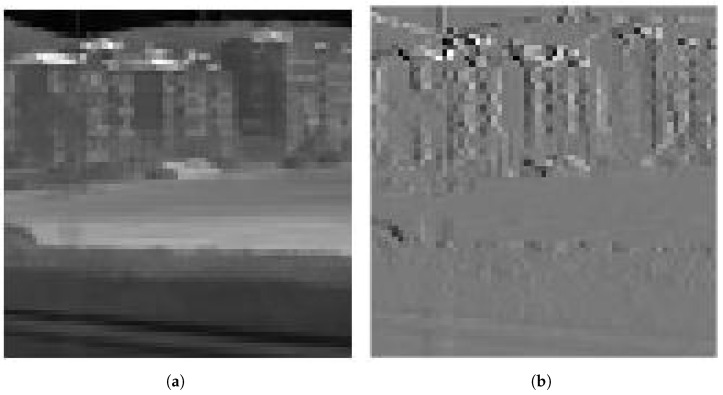
Illustration of DPCM-based redundancy reduction for the LL subband. (**a**) LL subband of the five-level DWT of Image A. (**b**) DPCM-based redundancy reduction for the LL subband.

**Figure 8:**
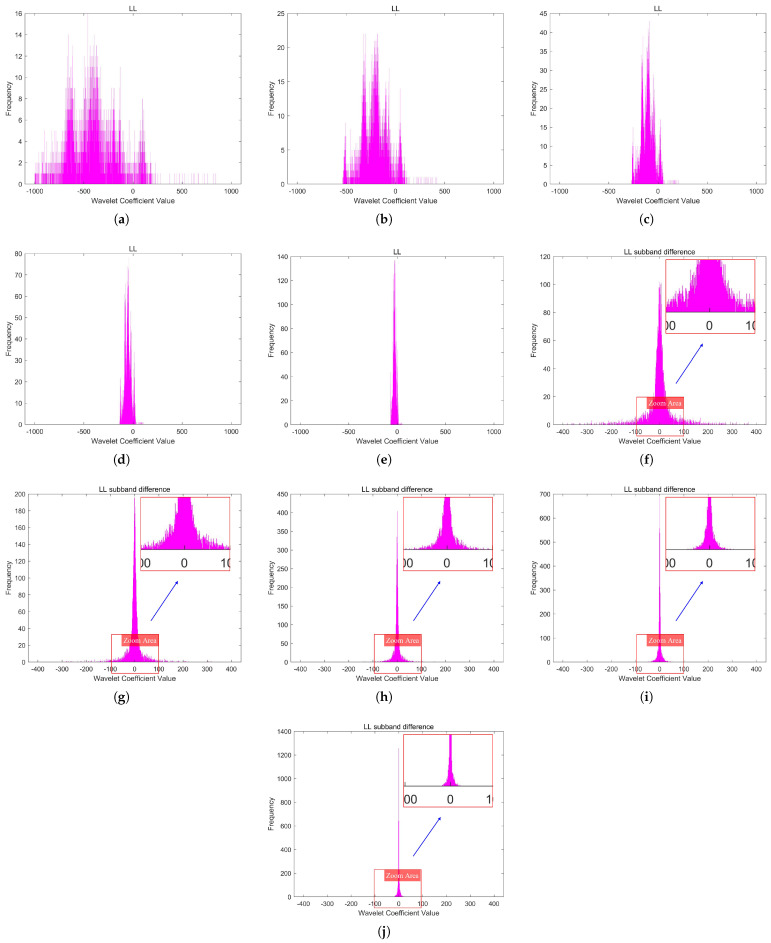
The coefficient histogram distributions of the LL subband and its DPCM redundancy-reduced version at different quantization levels. (**a**–**e**) depict the coefficient histograms of the LL subband of Image A, corresponding to quantization levels 1 to 5. (**f**–**j**) depict the coefficient histograms of its DPCM redundancy-reduced version, corresponding to quantization levels 1 to 5.

Figure 7 illustrates the DPCM redundancy reduction in the LL subband. The coefficient histogram distributions of the LL subband and its DPCM redundancy-reduced version at different quantization levels are shown in Figure 8.

#### 2.2.2. Probability Modeling of Quantized Wavelet Subbands

After the wavelet coefficients are quantized and spatial redundancy is removed, entropy coding is applied to form a compact bitstream. Common entropy coding methods include Huffman coding and arithmetic coding. Huffman coding enables encoding with complexity of O(1) after the code table is constructed, while arithmetic coding has higher complexity. In high-speed compression scenarios and on resource-constrained devices, Huffman coding is the preferred choice. Therefore, this paper uses Huffman coding for entropy encoding.

Entropy coding depends on the probability distribution of source symbols. Different subbands have distinct probability distributions; therefore, independently calculating symbol probabilities and constructing separate Huffman code tables for different subbands result in significant computational overhead, which in turn severely affects encoding speed.

Experiments have shown that the probability distributions of the wavelet high-frequency subbands and the LL subband after DPCM follow certain statistical patterns. Therefore, a probability model for each subband can be pre-established through extensive statistics, eliminating the need for symbol probability statistics during encoding. Based on the probability model, a Huffman code table can be pre-established, enabling encoding with a time complexity of O(1) through table lookups, significantly improving compression speed.

Conventional methods for constructing probability models typically involve selecting an appropriate known probability distribution, such as the exponential or Laplace distribution, based on the characteristics of the data distribution, and using Maximum Likelihood Estimation (MLE) to estimate the parameters. However, after DUSQ, the wavelet high-frequency subband coefficients exhibit significant sparsity with a large number of zeros, making it challenging for traditional probability distributions to accurately model the data. Therefore, this paper adopted a direct statistical approach to construct the probability model.

We applied a 5-level DWT to 53 infrared line-scan images and conducted a statistical analysis of the probability distributions of the quantized coefficients in each subband. The quantized coefficients in each subband were then sorted in descending order of probability. Finally, the probability distribution model was constructed based on the average probability distribution of the coefficients across the 53 images.

**Figure 9:**
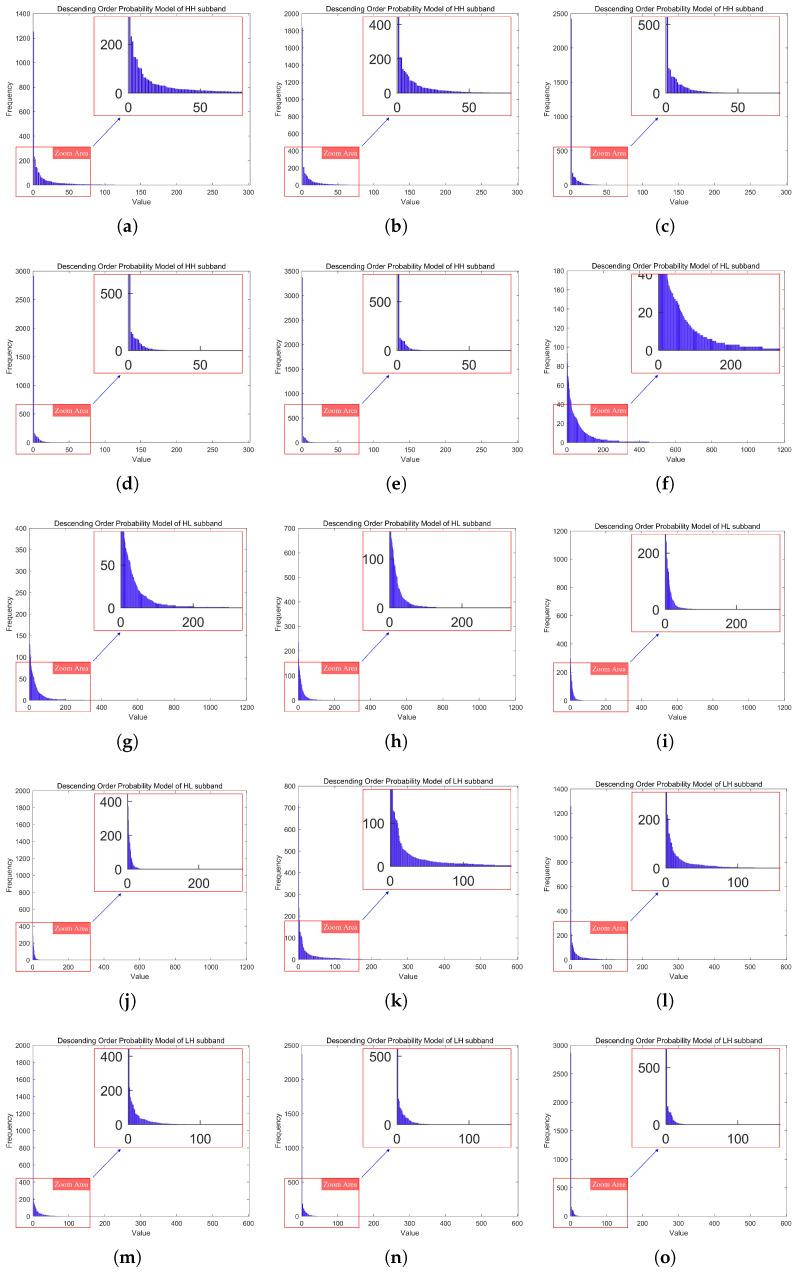
The probability models of the three fifth-level DWT high-frequency subbands (LH, HL, HH) at quantization levels 1–5. (**a**–**e**) depict the probability models of the HH subband at the fifth-level DWT, corresponding to quantization levels 1 to 5. (**f**–**j**) depict the probability models of the HL subband at the fifth-level DWT, corresponding to quantization levels 1 to 5. (**k**–**o**) depict the probability models of the LH subband at the fifth-level DWT, corresponding to quantization levels 1 to 5.

The probability models of the three fifth-level DWT high-frequency subbands (LH, HL, HH) at quantization levels 1–5 are shown in Figure 9.

**Figure 10:**
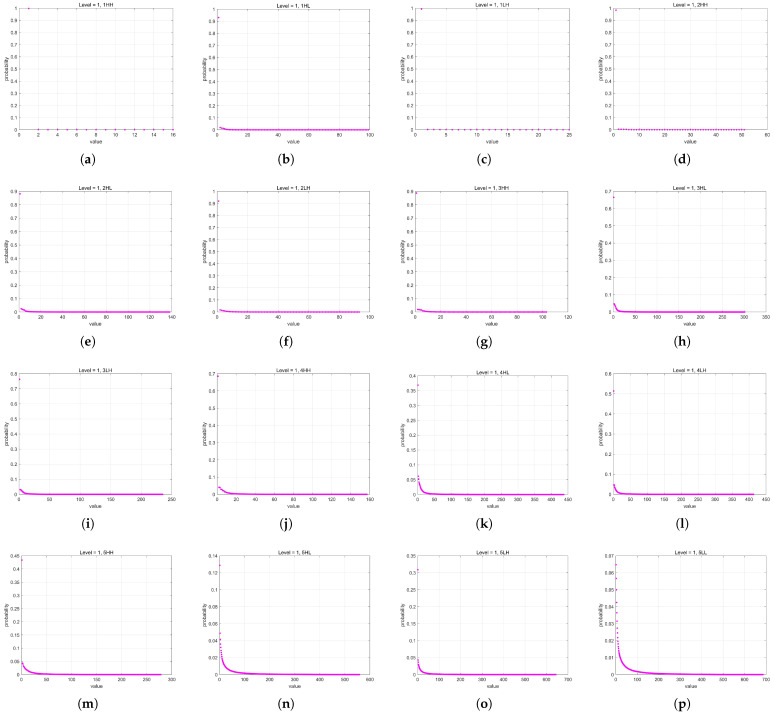
The probability models of all subbands in the five-level DWT when the quantization level is 1. (**a**–**o**) show the coefficient probability models of the (HH, HL, LH) subbands from the first to the fifth level of DWT in order. (**p**) depicts the coefficient probability model of the LL subband at the fifth level DWT.

The probability models of all subbands in the five-level DWT when the quantization level was 1 are shown in Figure 10.

Based on the probability models, Huffman code tables could be pre-established, thereby avoiding the need for probability statistics and code table construction for each subband during the encoding process. This enabled fast encoding through table lookup.

The established probability models usually have a certain boundary, but the probabilities outside the boundary are very small and can be neglected. Assuming that the boundary is [−pos,+pos], only 2·pos+1 symbols within this range are encoded. We adopted a method from our previous work [49] to handle symbols outside the boundary, where these symbols were encoded with the maximum code value and stored separately in a distinct storage space. During decoding, when the maximum code value was encountered, the symbols could be sequentially retrieved from the storage space. Since the probability of symbols outside the boundary was low, this had minimal impact on the compression performance.

Assuming that the code table $C=[c_{0},c_{1},c_{2},\ldots ,c_{2\cdot pos}]$ is constructed based on a probability model and Huffman coding rules, sorted in ascending order of code length, while the quantized coefficient probabilities follow the descending order represented as q=[0,1,−1,…,pos,−pos], the encoding rule can be expressed as follows:

(26) encode(q(index))=C(index)

where:

(27) $$index=\left\{\begin{array}{c} -2\cdot k,\mathrm{if}\;k\leq 0\\ 2\cdot k-1,\mathrm{if}\;k>0\\ 2\cdot pos+1,\mathrm{if}\;k>2\cdot pos \end{array}\right.$$

### 2.3. Run-Length-Enhanced Huffman Coding

#### 2.3.1. Analysis of the Limitations of Huffman Coding for Quantized Wavelet Coefficients with Sparse Distributions

**Figure 11:**
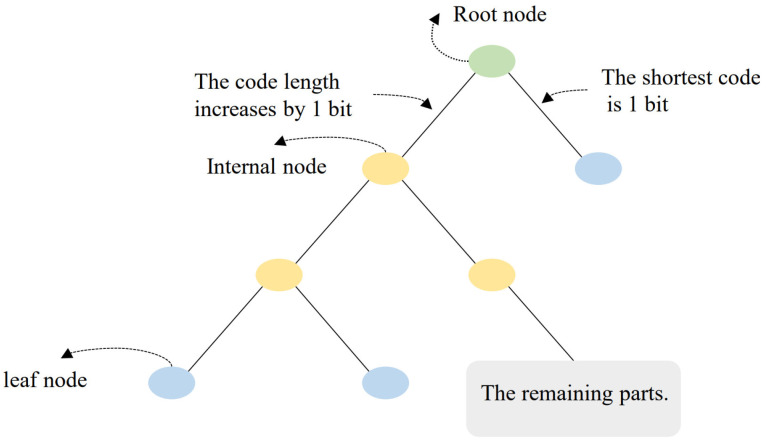
The mechanism of Huffman coding codeword length increase and the shortest code.

Huffman coding achieves the minimization of redundancy under the constraint of prefix codes. It is the closest to Shannon entropy among integer-length encodings and has simple and efficient encoding/decoding algorithms. Therefore, it is called “minimum redundancy coding” [39]. Huffman coding is based on constructing a binary tree, where each symbol corresponds to a leaf node. The length of the code is determined by the path length from the root node to the leaf node. Since a Huffman tree is a binary tree, each internal node extends downward to two child nodes. Every time a parent node extends to a child node, the path length increases by 1 bit, so the code length can only be an integer, as shown in Figure 11. While this restriction simplifies the encoding and decoding algorithms, it also reduces the coding efficiency of Huffman coding. Additionally, Huffman coding does not take into account the continuity of symbol distribution.

The drawbacks of Huffman coding lie in its allocation of integer-bit codes, which introduces imprecision, and its inability to account for the continuity of symbol distribution. Given a symbol set $X=[x_{0},x_{1},\ldots ,x_{n-1}]$ with occurrence probabilities $P=[p\left(x_{0}\right),p\left(x_{1}\right),\ldots ,p\left(x_{n-1}\right)]$, the self-information of a symbol $x_{i}$ is defined as:

(28) $$I\left(x_{i}\right)=-\log_{2}P\left(x_{i}\right)$$

The average self-information, also known as Shannon entropy, is defined as:

(29) $$H\left(X\right)=-\sum_{n}p\left(x_{i}\right)\cdot \log_{2}p\left(x_{i}\right)$$

In Huffman coding, let the code length of symbol $x_{i}$ be $n_{i}$. Then, $n_{i}$ must not be smaller than the self-information of $x_{i}$:

(30) $$n_{i}\geq -\log_{2}P\left(x_{i}\right)$$

After Huffman coding, the actual average code length is defined as:

(31) $$L_{avg}=\sum_{n}p\left(x_{i}\right)\cdot n_{i}$$

It follows that:

(32) $$L_{avg}\geq H\left(X\right)$$

H(X) is the theoretically shortest average encoding length. In Huffman coding, the actual encoding length $L_{avg}$ is close to H(X), but it cannot be smaller than H(X). They are equal only when the symbol probability is an integer power of 1/2. However, this is a rare and special case in image encoding.

After DUSQ, the frequency of zeros in the wavelet coefficients increases significantly, while the probabilities of other symbols remain low. In this case, zeros are assigned extremely short codes (typically 1 bit, such as “0”), while the others are assigned relatively longer codes. This allocation benefits overall compression efficiency since a higher proportion of zero values means that short codes are used more frequently, making the average code length closer to the Shannon entropy. However, since Huffman coding can only allocate integer bit codes, when more zeros occur, it aggravates the inaccurate code length allocation, resulting in a larger gap between the code length and the self-information of the symbol. In such cases, the gap is directly influenced by the probability distribution of the symbols.

Assuming a probability of 0, p(0)=0.8, the self-information is:

(33) $$I\left(0\right)=-\log_{2}\left(p\left(0\right)\right)=0.322\;\mathrm{bit}$$

The gap is:

(34) ΔI(0)=1−0.322=0.678bits

For a nonzero symbol $x_{i}$ with $p(x_{i})=0.01$, the self-information is:

(35) $$I\left(x_{i}\right)=-\log_{2}\left(p\left(x_{i}\right)\right)=6.644\;\mathrm{bit}$$

The gap is:

(36) $$\Delta I(x_{i})=7-6.644=0.356\;\mathrm{bits}$$

It is evident that when the probability of zero is high and the probabilities of other symbols are low, the Huffman encoding for zero deviates most from the self-information. Moreover, when the proportion of zeros in the data is large, this further aggravate the deviation between the average encoding length and the self-information. Therefore, in sparse quantized wavelet coefficients, reducing the encoding inaccuracy for zeros can significantly improve the effectiveness of Huffman coding.

#### 2.3.2. Run-Length-Enhanced Huffman Coding Algorithm

As the quantization level of wavelet coefficients increases, zeros appear more frequently, and their consecutive runs become longer. However, Huffman coding ignores the continuity of symbol distributions. Run-Length Coding (RLC) can effectively exploit this continuity and can be incorporated into Huffman coding to enhance compression efficiency.

Traditional methods typically separate Huffman coding and RLC, applying RLC to zeros while encoding other symbols using Huffman coding. However, this approach requires additional flag bits or prefixes in the encoded stream, which not only increases encoding overhead but also complicates the logic of both the encoder and decoder.

We propose a run-length-enhanced Huffman coding (Huf-RLC) method, which integrates RLC within Huffman coding. Specifically, we modified the shortest Huffman code by appending additional bits to record the run length of consecutive zeros. First, a codebook was generated based on the probability model. During encoding, when a zero was encountered, Huffman coding was applied, followed by a fixed number of bits representing the number of consecutive zeros. If the count exceeded the maximum recordable value, the remaining zeros were treated as a new sequence and encoded separately. For nonzero values, standard Huffman coding was used. During decoding, when encountering the zero-value encoding, the decoder could directly obtain the number of consecutive zeros from the encoded stream. The detailed description of the algorithm is presented in Algorithm 1.
**Algorithm 1** Run-length-enhanced Huffman coding.**Require:** $Quantized\text{\_}coeff=[q_{0},q_{1},q_{2},\cdots ,q_{n}]$, $C=[c_{0},c_{1},\cdots ,c_{2\cdot pos}]$**Ensure:** Bitstream 1:rlc=0 2:$\vec{B}$⇐0 3:Bitstream ⇐ [] 4:**for** each Quantized_coeff(i) in Quantized_coeff **do** 5:      **if** Quantized_coeff(i)=0 **then** 6:           rlc=rlc+1 7:           **if** $rlc==2^{\mathrm{bit}\_\mathrm{len}}-1$ **then** 8:                 index=0 9:                 Bitstream ⇐ Huffman_encode(C(index))10:                Bitstream ⇐ RLC_encode(rlc)11:                rlc=012:          **end if**13:     **else**14:          **if** rlc≠0 **then**15:                index=016:                Bitstream ⇐ Huffman_encode(C(index))17:                Bitstream ⇐ RLC_encode(rlc)18:                rlc=019:          **end if**20:          **if** Quantized_coeff(i)<0 **then**21:                index=2·(0−Quantized_coeff(i))+122:          **else**23:                index=2·Quantized_coeff(i)24:          **end if**25:          **if** index>2·pos **then**26:                index=2·pos+127:                $\vec{B}$⇐Quantized_coeff(i)28:          **end if**29:          Bitstream ⇐ Huffman_encode(C(index))30:     **end if**31:     Bitstream ⇐ encode($\vec{B}$)32:**end for**

To further enhance the theoretical completeness of the proposed Huf-RLC algorithm, the following provides an analysis of its time and space complexities.

Time Complexity Analysis: The algorithm processes the input array Quantized_coeff in a single pass, and each element only undergoes constant-time operations (such as counting, comparisons, index calculations, and table lookups), each of which has a time complexity of O(1). Since no element triggers more than a fixed number of such operations, the overall time complexity remains O(n), without introducing any higher-order polynomial overhead.

Space Complexity Analysis: Besides the input data, the algorithm utilizes a few constant-space variables (e.g., counter, index, and a temporary buffer), which means the extra space requirement is O(1). The main space usage comes from the output bitstream. Assuming that the average encoded length per input element is constant, the total space required for the bitstream scales linearly with the input size *n*, i.e., O(n).

In summary, the overall time complexity of the Huf-RLC algorithm is O(n), and the space complexity is O(n), making it efficient for processing large-scale data.

This method allows Huffman coding to take advantage of the continuity in the distribution of zeros, enabling Huffman coding to achieve fractional-bit encoding and improving its compression efficiency for sparse quantized wavelet coefficients. It is important to note that the number of bits used to record the consecutive zeros is related to the quantization level and the wavelet subbands, and the optimal bit length needs to be determined through experiments, which are presented in the results section.

### 2.4. Lossy Compression Algorithm Based on Huf-RLC and Wavelet Coefficient Probability Model

The previous sections discussed variable bitrate compression achieved by adjusting the quantization parameter Q. To simplify the probability model, the quantization parameter Q was categorized into five levels, with each level corresponding to specific probability distribution models and code tables. Different Q values within the same quantization level used the same probability models and code tables. Additionally, each wavelet subband in the image had its own probability model.

Since the probability of zeros is very high, their encoding method has a decisive impact on the final compression performance. Therefore, the number of bits used to record the count of zeros in the Huf-RLC significantly affects the compression efficiency. Thus, it is necessary to set the appropriate bit length for recording the number of zeros for different quantization levels and wavelet subbands.

**Figure 12:**
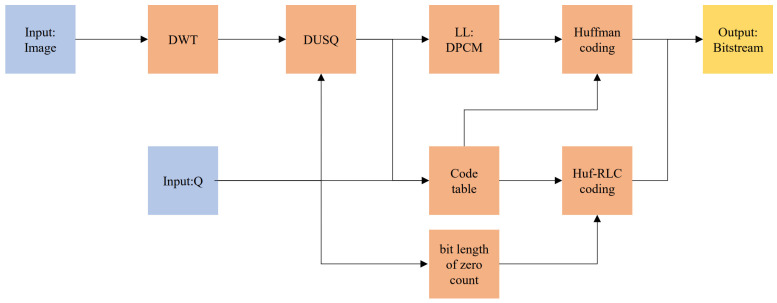
The framework of lossy compression algorithm based on Huf-RLC and wavelet coefficient probability model.

Based on the above analysis, the algorithm’s input parameters only included the quantization parameter Q and the input image. Adjusting Q allowed for compression at different bit rates. The quantization level could be determined based on the input parameter Q, thereby selecting the corresponding Huffman code table for the probability model of each subband at that level, as well as the number of bits for recording consecutive zeros. Thus, Huf-RLC could be directly carried out by looking up the code tables. The framework of the algorithm is shown in Figure 12.

## 3. Results

### 3.1. Selection of Optimal Run Length for Different Quantization Levels and Subbands

In Huf-RLC, the bit length used to record the number of consecutive zeros significantly impacts compression performance, especially when zeros occur with high probability. To determine the optimal bit length for encoding zero runs, experiments were conducted on five quantization levels of a five-level DWT across fifteen high-frequency subbands.

**Figure 13:**
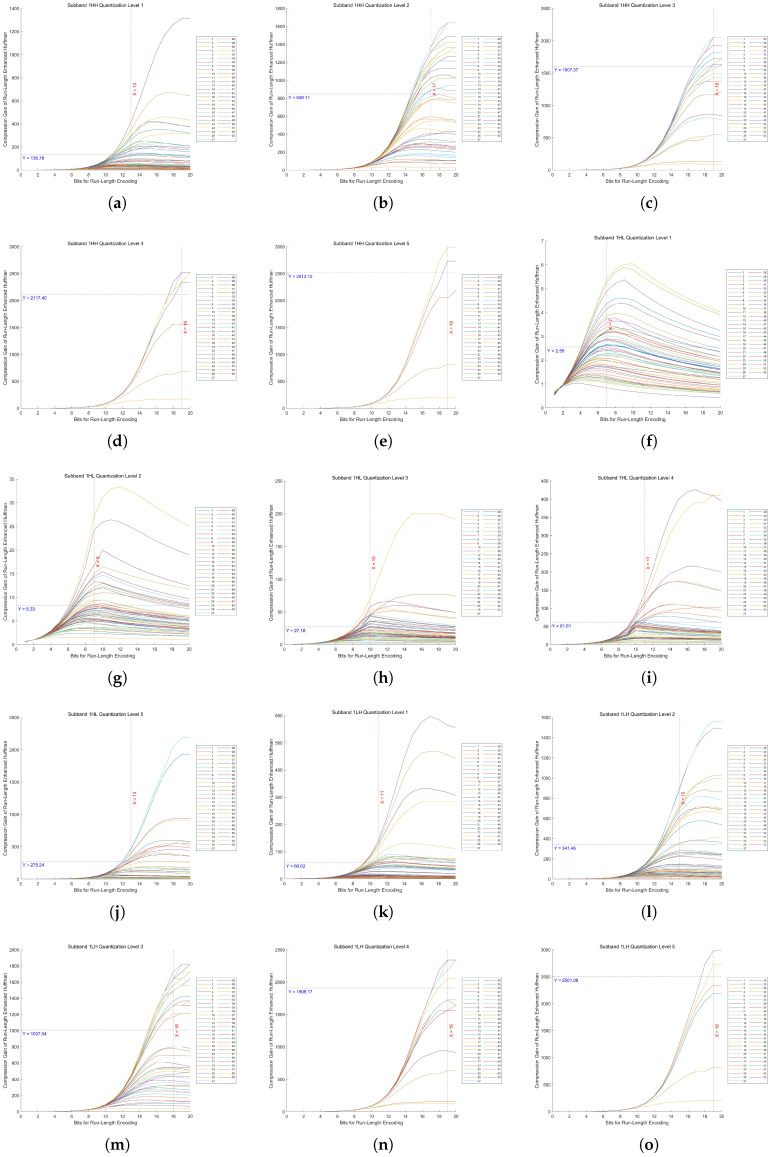
Compression gain vs. bit length in Huf-RLC for high-frequency subbands at decomposition level 1 across 53 images. (**a**–**e**) show the results of the HH subband at quantization levels 1 to 5, respectively. (**f**–**j**) show the results of the HL subband at quantization levels 1 to 5, respectively. (**k**–**o**) show the results of the LH subband at quantization levels 1 to 5, respectively.

**Figure A1:**
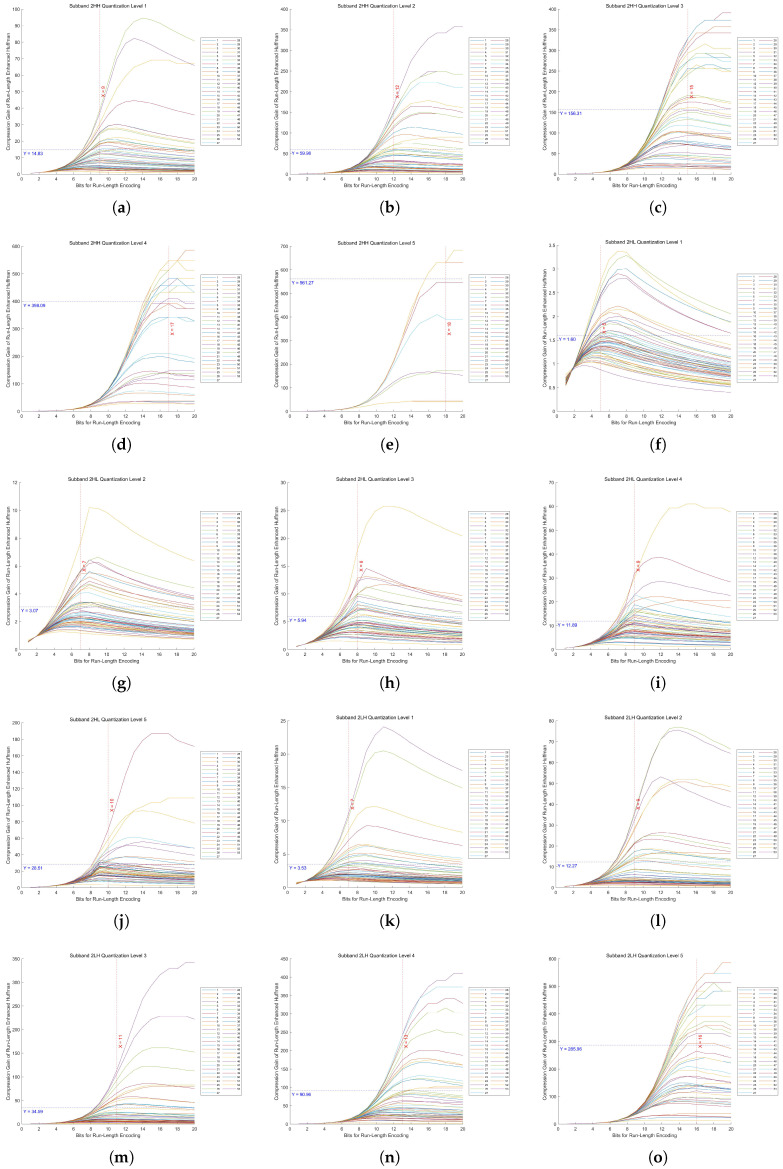
Compression gain vs. bit length in Huf-RLC for high-frequency subbands at decomposition level 2 across 53 images. (**a**–**e**) show the results of the HH subband at quantization levels 1 to 5, respectively. (**f**–**j**) show the results of the HL subband at quantization levels 1 to 5, respectively. (**k**–**o**) show the results of the LH subband at quantization levels 1 to 5, respectively.

**Figure A2:**
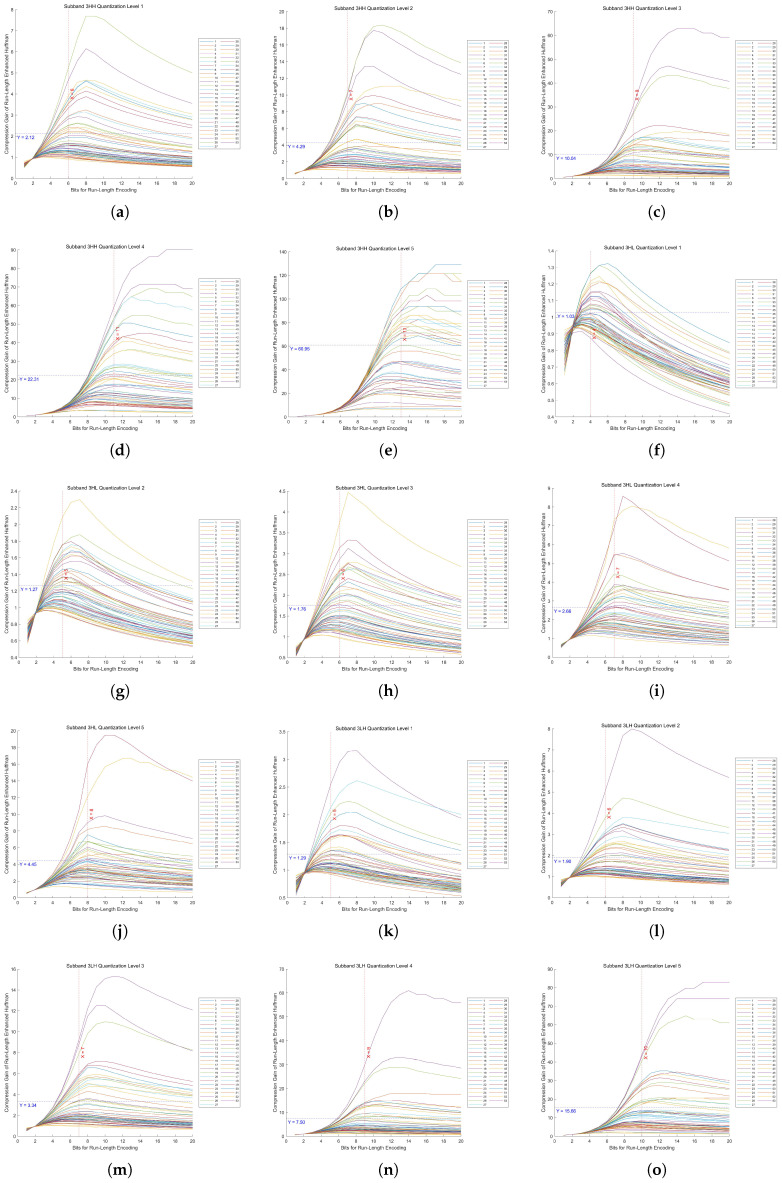
Compression gain vs. bit length in Huf-RLC for high-frequency subbands at decomposition level 3 across 53 images. (**a**–**e**) show the results of the HH subband at quantization levels 1 to 5, respectively. (**f**–**j**) show the results of the HL subband at quantization levels 1 to 5, respectively. (**k**–**o**) show the results of the LH subband at quantization levels 1 to 5, respectively.

**Figure A3:**
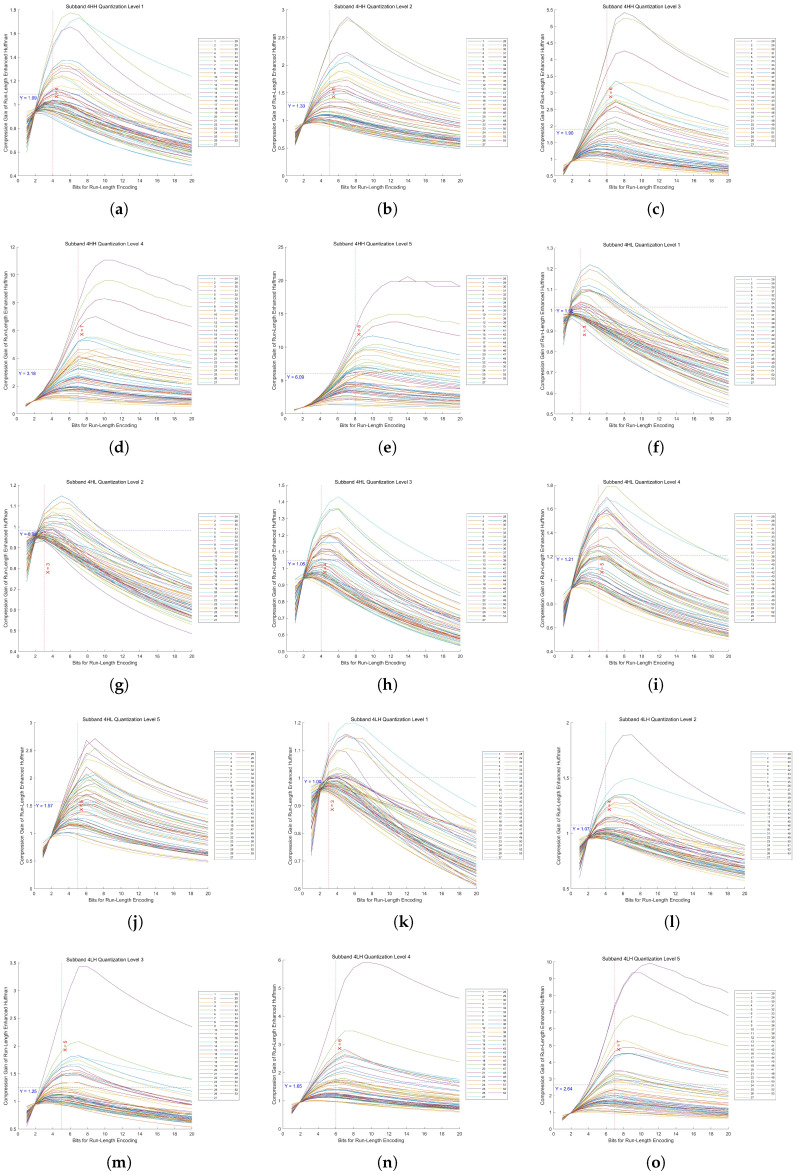
Compression gain vs. bit length in Huf-RLC for high-frequency subbands at decomposition level 4 across 53 images. (**a**–**e**) show the results of the HH subband at quantization levels 1 to 5, respectively. (**f**–**j**) show the results of the HL subband at quantization levels 1 to 5, respectively. (**k**–**o**) show the results of the LH subband at quantization levels 1 to 5, respectively.

**Figure A4:**
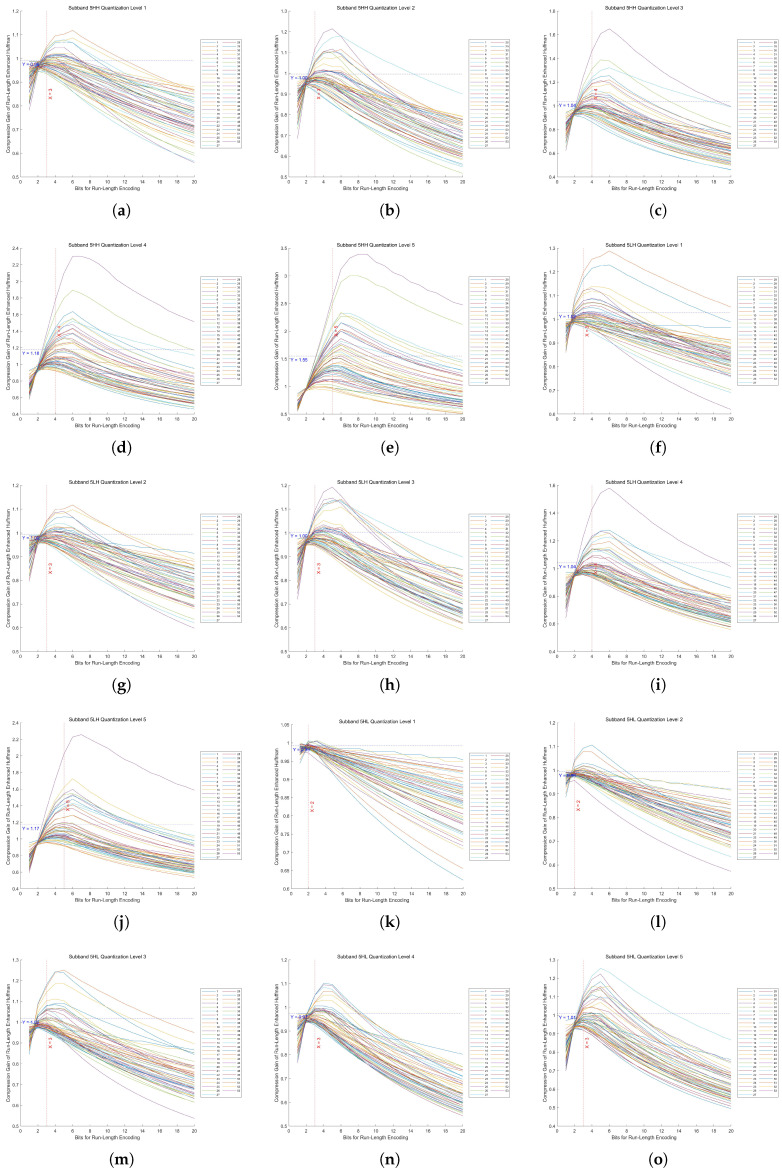
Compression gain vs. bit length in Huf-RLC for high-frequency subbands at decomposition level 5 across 53 images. (**a**–**e**) show the results of the HH subband at quantization levels 1 to 5, respectively. (**f**–**j**) show the results of the HL subband at quantization levels 1 to 5, respectively. (**k**–**o**) show the results of the LH subband at quantization levels 1 to 5, respectively.

By evaluating 53 images, we measured the compression gain, defined as the ratio of the Huf-RLC compression ratio to the standard Huffman compression ratio, across bit lengths ranging from 1 to 20. The experimental results for the high-frequency subbands at decomposition level 1 in a five-level DWT, under quantization levels 1 to 5, are shown in Figure 13. The results for decomposition levels 2 to 5 are presented in Appendix A.2 in Figure A1, Figure A2, Figure A3, and Figure A4, respectively.

As proven in our theoretical analysis (Appendix A.1), there exists a unique optimal bit length $k^{*}$ for run-length encoding of zeros in quantized high-frequency wavelet subbands. From these figures, we can observe that the distribution of the 53 values of $k^{*}$ is concentrated around a certain value, and the distribution may approximate a normal distribution. Therefore, using the mean as an estimate of the overall optimal value is a reasonable and effective approach.

In these figures, the x-axis represents the bit length used for encoding zero runs, while the y-axis denotes the compression gain. Five quantization levels were analyzed, with 53 curves corresponding to individual images. Each curve is annotated with the mean of all y-values at its maximum point, along with the rounded mean of the corresponding x-values. This rounded x-value represents the optimal bit length for recording the number of consecutive zeros.

Furthermore, Huf-RLC was applied to a subband only if the compression gain exceeded two (i.e., y > 2); otherwise, standard Huffman coding was used.

### 3.2. Comparison of Performance with JPEG and JPEG2000 at Different Bitrates

In lossy image compression, PSNR and SSIM are metrics used to evaluate the quality of compressed images. PSNR primarily measures pixel-wise errors, while SSIM focuses more on structure, brightness, and contrast, making it better at reflecting human visual perception.

PSNR is defined as follows:

(37) $$PSNR=10\log_{10}\left(\frac{MAX^{2}}{MSE}\right)$$

(38) $$MSE=\frac{1}{m\times n}\sum_{i=1}^{m}\sum_{j=1}^{n}{\left(X\left(i,j\right)-Y\left(i,j\right)\right)}^{2}$$

where MAX is the maximum pixel value (255 for the eight-bit images in this paper). The original image *X* is of size m×n, and the compressed image *Y* is also of size m×n.

SSIM is defined as follows:

(39) $$SSIM\left(X,Y\right)=\frac{\left(2\mu_{X}\mu_{Y}+C_{1}\right)\left(2\sigma_{XY}+C_{2}\right)}{\left(\mu_{X}^{2}+\mu_{Y}^{2}+C_{1}\right)\left(\sigma_{X}^{2}+\sigma_{Y}^{2}+C_{2}\right)}$$

where $\mu_{X},\mu_{Y}$ are the means of images *X* and *Y* (representing brightness). $\sigma_{X}^{2},\sigma_{Y}^{2}$ are the variances (representing contrast). $\sigma_{XY}$ is the covariance (representing structural information). $C_{1},C_{2}$ are stability constants to prevent division by zero.

BPP (Bits Per Pixel) is a key metric for evaluating image compression quality and efficiency. It indicates the average number of bits assigned to each pixel in the compressed image and helps quantify the trade-off between compression ratio and image quality.

BPP is defined as follows:

(40) $$BPP=\frac{S_{\mathrm{compressed}}}{m\times n}$$

where $S_{\mathrm{compressed}}$ is the compressed image size (in bits).

Since the probability models was derived from 53 images, additional images were required to validate the generalizability of the algorithm, similar to the training and testing sets in deep learning. In this study, 53 images were used to construct the training set, while another 27 images were collected from different working scenarios of infrared line-scan detectors to form the test set.

**Figure 14:**
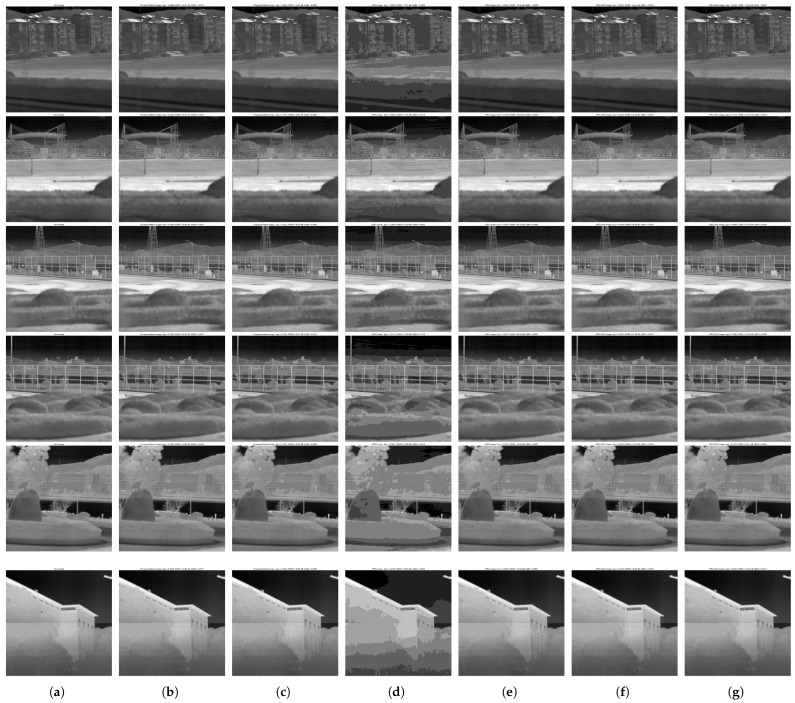
Comparison of performance with JPEG and JPEG2000 at different bitrates. (**a**) Initial images. (**b**) Reconstructed images at low bitrate (proposed method). (**c**) Reconstructed images at high bitrate (proposed method). (**d**) Reconstructed images at low bitrate (JPEG). (**e**) Reconstructed images at high bitrate (JPEG). (**f**) Reconstructed images at low bitrate (JPEG2000). (**g**) Reconstructed images at high bitrate (JPEG2000).

In the experiment, 3 images were selected from the 53 images in the training set and 3 images from the 27 images in the test set to compare the performance of the proposed method, JPEG, and JPEG2000 at different bitrates. The specific bitrates were categorized into low bitrate under the first quantization level and high bitrate under the fifth quantization level. The experimental results are shown in Figure 14, with detailed BPP, PSNR, and SSIM statistics provided in Table 5.

JPEG2000 performed well under low bitrate conditions. The above test results and their averages showed that the proposed method maintained a PSNR of 40.05 dB and an SSIM of 0.9649 at a low bitrate (BPP approximately 0.0380). Compared to JPEG2000, the PSNR loss did not exceed 2.67 dB, and the SSIM loss did not exceed 0.008. However, JPEG, even at a relatively higher BPP (approximately 0.0420), exhibited noticeable block effects, with a PSNR loss of 14.28 dB and an SSIM loss of 0.1165.

### 3.3. Rate-Distortion Curves and Compression Speed Evaluation

**Figure 15:**
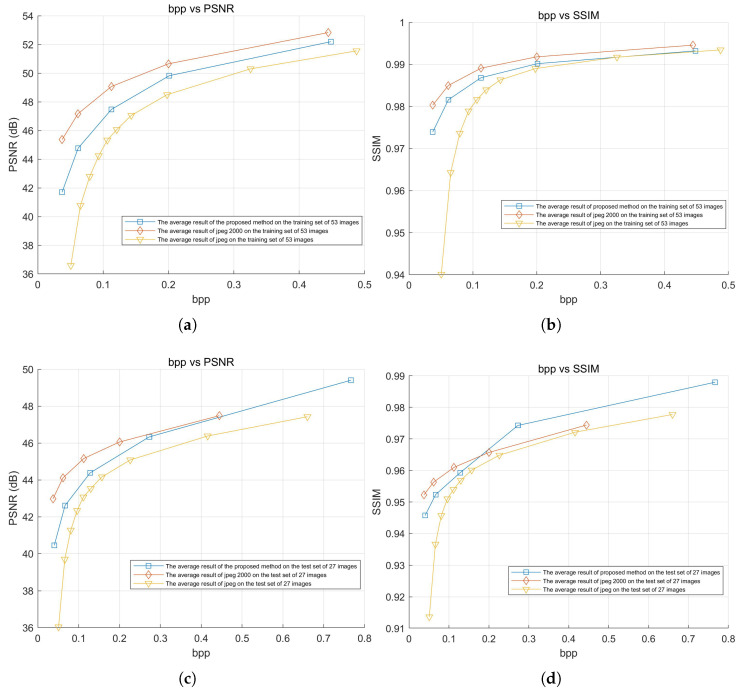
Rate–distortion curve. (**a**) Average PSNR vs. BPP (training set, 53 images). (**b**) Average SSIM vs. BPP (training set, 53 images). (**c**) Average PSNR vs. BPP (test set, 27 images). (**d**) Average SSIM vs. BPP (test set, 27 images).

The experiment tested the compression efficiency of the proposed method, JPEG, and JPEG2000 on both the training and test sets. The average PSNR and SSIM results for all images in the dataset at different bitrates were used to plot the curves shown in Figure 15. The average results of these curves, along with detailed numerical comparisons at specific points, are summarized in Table 6. Additionally, the speed of the proposed method, JPEG, and JPEG2000 was also tested and is presented in Table 7.

The experimental results show that, in terms of rate–distortion performance, the proposed method outperformed JPEG and was close to JPEG2000. In the average test results of the 53 training images, the PSNR loss compared to JPEG2000 was only −1.825 dB, and the SSIM loss was only −0.003, while the PSNR loss for JPEG reached −3.699 dB, and the SSIM loss was approximately −0.00985. In the average test results of the 27 test images, the PSNR loss compared to JPEG2000 was only −0.520 dB, and the SSIM improved by 0.002, while JPEG’s PSNR loss reached −2.257 dB, and the SSIM loss was approximately −0.00865.

Especially under low bitrate conditions, the proposed method still maintained a small gap with JPEG2000, while JPEG showed severe degradation. For the training set at a low bitrate, the PSNR loss compared to JPEG2000 was only −3.665 dB, with an SSIM loss of −0.00640, whereas JPEG showed a greater PSNR loss of −8.779 dB and an SSIM loss of approximately −0.04022. For the test set at a low bitrate, the PSNR loss compared to JPEG2000 was −2.522 dB, with an SSIM loss of −0.00652, while JPEG exhibited a higher PSNR loss of −6.934 dB, and an SSIM loss of approximately −0.03859.

JPEG2000 adopts MQ arithmetic coding, a context-based binary entropy encoder that operates at the bit level. It employs a two-level lookup table based on context labels (CX) and binary decisions (D) to adaptively estimate symbol probabilities. This fine-grained and highly adaptive coding allows the encoder to approach the theoretical entropy limit and achieve high compression efficiency. However, the computational complexity introduced by bit-level processing and context modeling may limit its suitability for real-time or resource-constrained scenarios. In contrast, the proposed Huf-RLC method performs symbol-level entropy coding using a much simpler structure. While this sacrifices some compression performance, it significantly reduces computational overhead and offers much faster encoding, making it more appropriate for speed-critical applications.

The speed test results showed that the proposed method achieved a speed of 28.944 MB/s, which was approximately 3.155 times faster than JPEG and 2.049 times faster than JPEG2000.

We implemented the DWT in the same manner as OpenJPEG, utilizing SIMD technology to ensure the efficiency of the DWT implementation. Despite this, the compression speed of our method outperformed OpenJPEG, primarily due to the advantages of our proposed entropy coding method, Huf-RLC. Notably, Huf-RLC was tested using a single core and a single thread, without any SIMD optimizations or acceleration techniques. This demonstrates the speed advantages of Huf-RLC, even in the absence of optimizations.

The image encoding time measurement procedure involved testing the encoding speed of each image individually, with the average encoding time calculated across all images. This was performed using a Windows batch script that processed each image one by one. The testing was conducted without any specific bulk encoding mechanism, ensuring that each image was encoded separately.

The above experimental results were conducted on an experimental platform with a 12th Gen Intel(R) Core(TM) i7-12700H, a 20-core CPU at 2.30 GHz, and 16 GB of RAM.

Deep learning-based compression methods that currently outperform JPEG2000 in terms of compression efficiency generally come with increased computational complexity. However, for applications where compression speed is critical, such as high-speed scenarios, our method offers a significant advantage due to faster encoding. As a result, comparisons with deep learning-based methods were not included in the experiments.

## 4. Conclusions

This paper proposed a novel lossy compression algorithm for infrared images, based on a quantized wavelet coefficient probability model and run-length-enhanced Huffman coding (Huf-RLC). The algorithm aimed to achieve faster compression speeds than JPEG2000, while maintaining compression efficiency close to that of JPEG2000, making it suitable for applications where speed is a key requirement. In the experiments, infrared images collected from different experimental scenarios using an infrared line-scanning detector were used to form a training set of 53 images and a test set of 27 images. The training set was used to build the quantized wavelet coefficient probability model, specifically constructing probability models for 16 subbands of a five-level DWT at five quantization levels, thus avoiding the complex statistics required for building coding tables for each subband coefficient during entropy coding. Based on the sparse characteristics of the subband coefficients in the line-scanned infrared images at different quantization levels, a run-length-enhanced Huffman coding was further designed. In the experiments, we analyzed in detail the effect of run length on compression gain across different quantization levels and subbands and determined the optimal run length for each probability model to achieve optimal compression performance.

Experimental results showed that the proposed method outperformed JPEG in terms of PSNR and SSIM, and when compared to JPEG2000, it ensured that the performance loss remained within a small range. Especially under low bitrate conditions, the proposed method maintained a small gap with JPEG2000, while JPEG showed significant blocking artifacts. Speed test results on an experimental platform with a 12th Gen Intel(R) Core(TM) i7-12700H (2.30 GHz, approximately 20-core CPU, 16 GB RAM) showed that the proposed method achieved compression speeds 3.155 times faster than JPEG2000 and 2.049 times faster than JPEG, providing an ideal solution for lossy compression applications that require both compression speed and image quality.

## Figures and Tables

**Table 1:** The filter coefficients of the Daubechies 9/7 DWT.

Daubechies 9/7	Analysis Filter Coefficients		Synthesis Filter Coefficients	
*n*	h[n]	g[n]	$\tilde{h}\left[n\right]$	$\tilde{g}\left[n\right]$
0	0.6029490182364	1.115087052457	1.1150870524570	0.6029490182364
±1	0.2668641184429	−0.5912717631142	0.5912717631142	−0.2668641184429
±2	−0.0782232665290	−0.0575435262285	−0.0575435262285	−0.0782232665290
±3	−0.0168641184429	0.0912717631143	−0.0912717631143	0.0168641184429
±4	0.0267487574108	-	-	0.0267487574108

**Table 2:** The parameters of the lifting-based Daubechies 9/7 DWT.

α	β	γ	δ	ξ
−1.586134342	−0.05298011854	0.8829110762	0.4435068522	1.149604398

**Table 3:** Division of quantization levels.

Q	Level
(0,2]	5
(2,4]	4
(4,8]	3
(8,16]	2
(16,32]	1

**Table 4:** Entropy of subbands before and after DPCM.

Image A		1-Level DWT	2-Level DWT	3-Level DWT	4-Level DWT	5-Level DWT
LH	initial	0.1328	1.0354	2.5447	4.3155	6.1932
	DPCM1 ^1^	0.2107	1.4587	3.2105	5.1151	6.9936
	DPCM2 ^2^	0.1442	1.1379	2.7488	4.6707	6.5551
HL	initial	0.3513	1.0436	3.1289	5.5041	7.5204
	DPCM1	0.3986	1.0571	3.0345	5.1213	6.9991
	DPCM2	0.4765	1.4886	3.9721	6.4089	8.2336
HH	initial	0.0035	0.1981	1.4318	3.1328	5.0752
	DPCM1	0.0064	0.3101	1.9424	3.8195	5.8301
	DPCM2	0.0060	0.3114	1.9698	3.8914	5.8806
LL	initial	-	-	-	-	9.7194
	DPCM1	-	-	-	-	**7.3093**
	DPCM2	-	-	-	-	**8.5251**

^1^ “DPCM1” represents column-wise DPCM; ^2^ “DPCM2” represents row-wise DPCM.

**Table 5:** Performance comparison (BPP, PSNR, SSIM) at different bitrates.

Image	Method	BPP	PSNR	SSIM	BPP	PSNR	SSIM
1	JPEG	0.0387	27.37	0.8456	0.5282	50.84	0.9928
	JPEG2000	0.0381	43.41	0.9793	0.5000	52.4	0.9947
	Proposed	0.0380	40.57	0.9704	0.4983	51.63	0.9928
2	JPEG	0.0442	31.24	0.8715	0.7234	49.56	0.9903
	JPEG2000	0.0442	42.98	0.9729	0.7273	50.97	0.9926
	Proposed	0.0440	39.91	0.9618	0.7178	49.87	0.9895
3	JPEG	0.0465	31.03	0.8665	0.8340	49.26	0.9899
	JPEG2000	0.0435	42.00	0.9695	0.8889	50.78	0.9924
	Proposed	0.0434	39.64	0.9608	0.8353	49.46	0.9890
4	JPEG	0.0413	27.96	0.7719	0.8577	48.92	0.9887
	JPEG2000	0.0354	41.35	0.9621	1.0000	50.59	0.9920
	Proposed	0.0353	39.53	0.9537	0.9193	49.12	0.9879
5	JPEG	0.0387	27.96	0.8448	0.7216	49.56	0.9897
	JPEG2000	0.0327	42.72	0.9708	0.7273	50.81	0.9922
	Proposed	0.0326	40.55	0.9648	0.7037	49.73	0.9889
6	JPEG	0.0425	28.25	0.8902	0.5488	49.89	0.9897
	JPEG2000	0.0346	46.58	0.9828	0.5333	50.82	0.9917
	Proposed	0.0345	42.80	0.9781	0.5302	49.88	0.9886
Mean	JPEG	0.0420	28.97	0.8484	0.702	49.67	0.9901
	JPEG2000	0.0380	43.17	0.9729	0.729	51.06	0.9926
	Proposed	0.0380	40.50	0.9649	0.701	49.95	0.9894

**Table 6:** Performance comparison (BPP, PSNR, SSIM) at different bitrates.

Compared to JPEG2000		Proposed PSNR	Proposed SSIM	JPEG PSNR	JPEG SSIM
	Mean	−1.825	−0.00300	−3.699	−0.00985
Training set	Low BPP	−3.665	−0.00640	−8.779	−0.04022
	High BPP	−0.637	−0.00136	−1.278	−0.00113
	Mean	−0.520	0.00200	−2.257	−0.00865
Test set	Low BPP	−2.522	−0.00652	−6.934	−0.03859

**Table 7:** Compression speed test results.

Method	JPEG	JPEG2000	Proposed
**Speed (MB/S)**	14.153	9.174	28.944

## Data Availability

The data are available on request from the corresponding author.

## Appendix A

### Appendix A.1. Existence and Uniqueness of Optimal Fixed-Bit-Length Encoding

**Theorem** **A1.**
*Existence and Uniqueness of Optimal Fixed-Bit-Length Encoding.*

Let ${\left\{L_{i}\right\}}_{i=1}^{M}$ be a sequence of run lengths of zeros in a quantized high-frequency wavelet subband, where $L_{i}\in N^{+}$. If each run length is encoded using a fixed *k*-bit representation (with a maximum representable run length of $2^{k}-1$), then the total encoding cost function Total(k) admits a unique globally optimal bit length $k^{*}$, such that:

$$k^{*}=\arg\min_{k\in N^{+}}\mathrm{Total}\left(k\right),\;\mathrm{and}\;\forall k\neq k^{*},\;\mathrm{Total}\left(k\right)>\mathrm{Total}\left(k^{*}\right).$$

**Proof of Theorem 1.** The proof is as follows. Step 1: definitions and notation:

- **Run-length splitting rule**: for a run length $L_{i}>2^{k}-1$, split it into $n_{i}\left(k\right)=\left\lceil L_{i}/\left(2^{k}-1\right)\right\rceil$ segments, each encoded with *k* bits.
- **Total encoding cost**:
  $$\mathrm{Total}\left(k\right)=k\cdot \sum_{i=1}^{M}n_{i}\left(k\right).$$
- **Per-run cost function**: for a single $L_{i}$, define $f_{i}\left(k\right)=k\cdot \left\lceil L_{i}/\left(2^{k}-1\right)\right\rceil$, yielding $\mathrm{Total}\left(k\right)=\sum_{i=1}^{M}f_{i}\left(k\right)$.

Step 2: unimodality of $f_{i}\left(k\right)$ For any $L_{i}$, analyze $f_{i}\left(k\right)$ as *k* increases:

1. **Decreasing phase**: For a small *k*, $2^{k}-1$ grows exponentially, causing $\left\lceil L_{i}/\left(2^{k}-1\right)\right\rceil$ to decrease rapidly. The linear increase in *k* is dominated by the reduction in splits, so $f_{i}\left(k\right)$ decreases.
2. **Minimum point**: There exists $k_{i}^{*}$ where $f_{i}\left(k_{i}^{*}\right)$ is minimized. Beyond $k_{i}^{*}$, further increases in *k* outweigh the benefits of fewer splits.
3. **Increasing phase**: for $k\geq k_{i}^{*}$, $\lceil L_{i}/\left(2^{k}-1\right)\rceil =1$, and $f_{i}\left(k\right)=k$ grows linearly.

Thus, $f_{i}\left(k\right)$ is *unimodal* with a unique minimum. Step 3: unimodality of Total(k) To rigorously prove the unimodality of Total(k), we analyze its discrete derivative (forward difference). Let ΔTotal(k)=Total(k+1)−Total(k). We show that ΔTotal(k) changes sign at most once, from negative to positive, ensuring a unique minimum.

1. **Per-run forward difference**: For each $f_{i}\left(k\right)$, define its forward difference:
  $$\Delta f_{i}\left(k\right)=f_{i}\left(k+1\right)-f_{i}\left(k\right).$$
  From the unimodality of $f_{i}\left(k\right)$, there exists a critical $k_{i}^{*}$ such that:
  $$\Delta f_{i}\left(k\right)\left\{\begin{array}{cc} <0, & \mathrm{if}\;k<k_{i}^{*},\\ \geq 0, & \mathrm{if}\;k\geq k_{i}^{*}. \end{array}\right.$$
2. **Total forward difference**: The total difference is the sum of individual differences:
  $$\Delta \mathrm{Total}\left(k\right)=\sum_{i=1}^{M}\Delta f_{i}\left(k\right).$$
  Define two index sets at any *k*:
  $$D\left(k\right)=\left\{i|k<k_{i}^{*}\right\}\;\left(\mathrm{indices}\;\mathrm{where}\;\Delta f_{i}\left(k\right)<0\right),$$
  $$I\left(k\right)=\left\{i|k\geq k_{i}^{*}\right\}\;\left(\mathrm{indices}\;\mathrm{where}\;\Delta f_{i}\left(k\right)\geq 0\right).$$
  Then,
  $$\Delta \mathrm{Total}\left(k\right)=\underset{\mathrm{Negative}}{\underbrace{\sum_{i\in D(k)}\Delta f_{i}\left(k\right)}}+\underset{\mathrm{Non}\text{-}\mathrm{negative}}{\underbrace{\sum_{i\in I(k)}\Delta f_{i}\left(k\right)}}.$$
3. **Monotonicity of ΔTotal(k)**: As *k* increases:
  - D(k) shrinks because $k_{i}^{*}$ are fixed.
  - I(k) expands.
  Thus, the negative term $\sum_{i\in D(k)}\Delta f_{i}\left(k\right)$ decreases in magnitude, while the non-negative term $\sum_{i\in I(k)}\Delta f_{i}\left(k\right)$ increases. Consequently,
  - For $k<\min_{i}k_{i}^{*}$: D(k)={1,2,⋯,M}, so ΔTotal(k)<0.
  - For $k\geq \max_{i}k_{i}^{*}$: I(k)={1,2,⋯,M}, so ΔTotal(k)≥0.
  - For $k\in (\min_{i}k_{i}^{*},\max_{i}k_{i}^{*})$: the transition from ΔTotal(k)<0 to ΔTotal(k)≥0 occurs exactly once.

This implies Total(k) strictly decreases until $k^{*}$, then strictly increases, proving the unimodality and uniqueness of $k^{*}$.  □

### Appendix A.2. Compression Gain Results for Decomposition Levels 2–5

This appendix presents the experimental results of compression gain for the high-frequency subbands at decomposition levels 2 to 5 in a five-level DWT. For each level, 53 images were evaluated across bit lengths from 1 to 20 and five quantization levels.
